# Phenotypic senescence in a natural insect population

**DOI:** 10.1002/ece3.9668

**Published:** 2022-12-29

**Authors:** Kata Pásztor, Ádám Kőrösi, Ádám Gór, Viktor Szigeti, Flóra Vajna, János Kis

**Affiliations:** ^1^ Doctoral School of Biological Sciences Hungarian University of Agriculture and Life Sciences Gödöllő Hungary; ^2^ MTA‐ELTE‐MTM Ecology Research Group Budapest Hungary; ^3^ Büro Geyer und Dolek Wörthsee Germany; ^4^ The Doctoral School of Veterinary Science University of Veterinary Medicine Budapest Budapest Hungary; ^5^ Lendület Ecosystem Services Research Group Institute of Ecology and Botany, Centre for Ecological Research Vácrátót Hungary; ^6^ Department of Ecology, Institute for Biology University of Veterinary Medicine Budapest Budapest Hungary

**Keywords:** body size variation, in vivo measurements, insect population, mark‐recapture, natural long‐term study, phenotypic senescence

## Abstract

Senescence seems to be universal in living organisms and plays a major role in life‐history strategies. Phenotypic senescence, the decline of body condition and/or performance with age, is a largely understudied component of senescence in natural insect populations, although it would be important to understand how and why insects age under natural conditions. We aimed (i) to investigate how body mass and thorax width change with age in a natural population of the univoltine Clouded Apollo butterfly (*Parnassius mnemosyne*, Lepidoptera: Papilionidae) and (ii) to assess the relationship of this change with sex and wing length. We studied a population between 2014 and 2020 using mark‐recapture during the whole flight period each year. Repeated measurements of body mass and thorax width and single measurements of wing length were performed on marked individuals. We analyzed body mass and thorax width change with age (days since marking), wing length, and the date of the first capture. Both body mass and thorax width declined nonlinearly with age. Individuals appearing earlier in the flight period had significantly higher initial body mass and thorax width and their body mass declined faster than later ones. Initial body sizes of females were higher, but males' body sizes decreased slower. Initial thorax width showed higher annual variation than body mass. To our best knowledge, this is the first study that revealed phenotypic senescence in a natural butterfly population, using in vivo measurements. We found sexual differences in the rate of phenotypic senescence. Despite the annual variation of initial body sizes, the rate of senescence did not vary considerably across the years.

## Graphical abstract

Phenotypic senescence is a largely understudied component of senescence in natural insect populations. In a wild Clouded Apollo population investigated by us, both body mass and thorax width declined nonlinearly with age. Individuals appearing earlier in the flight period had significantly higher initial body mass and thorax width, and their body mass declined faster than later ones. Initial body sizes of females were higher, but males' body sizes decreased slower. Initial thorax width showed higher annual variation than body mass.

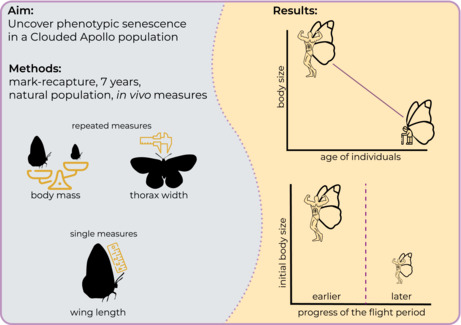

## 1 INTRODUCTION

Senescence, the degradation of physiological function and the decline of fitness with age, (Rose, 1994) is found in all living organisms. Environmental stressors, such as extreme heat, may accelerate aging, thus impacting demography and, ultimately, the viability of populations (Brunet‐Rossinni & Austad, 2005). Rapid human‐induced changes in natural habitats impose evolutionarily new stressors that may have detrimental consequences to organisms, and accelerated aging due to stressors may be an important factor in population decline. Senescence has been revealed in natural populations of a wide range of vertebrates (for a review of mammals and birds see: Nussey et al., 2013); however, our knowledge is scarce about senescence and life span in natural populations of invertebrates, which are mostly studied in the laboratory (Nussey et al., 2013; Zajitschek & Bonduriansky, 2014). Generalizations across taxa are questionable because of differences in physiology, reproduction, selection on late‐life performance (Zajitschek et al., 2020), or trade‐offs between senescence and other life‐history traits (Fry, 2008).

We distinguish actuarial senescence (decreasing survival rate), reproductive senescence (reduction of reproductive success), and phenotypic senescence (phenotypical recession) (Partridge & Barton, 1996). Actuarial and reproductive senescence in a wild insect population was demonstrated for the first time relatively recently in antler flies (Bonduriansky & Brassil, 2002). Since then, further studies have revealed an age‐related increase in mortality rates in wild insect populations including honey bees (Dukas, 2008a, 2008b), antler flies (Bonduriansky & Brassil, 2002, 2005; Mautz et al., 2019), field crickets (Rodríguez‐Muñoz et al., 2019), damselflies (Sherratt et al., 2010), dragonflies (Sherratt et al., 2011), mosquitoes (Ryan et al., 2015), and butterflies (Carroll & Sherratt, 2017; Sielezniew et al., 2020), but physiological changes underlying senescence are still understudied (Nussey et al., 2008).

By studying phenotypic senescence, measured through the disintegration of the body, we can broaden our knowledge about the differences in individuals' life‐history strategies within populations (Fisher et al., 2018; Réale et al., 2010) and the age‐related decline in performance. Life‐history traits (e.g., age and size at reproduction, fecundity, growth rate) describe the major features of an organism's life cycle (Stearns, 1992). The rate of senescence may be affected by the resource allocation between maintaining body condition at an older age (phenotypic and actuarial senescence) or increasing reproductive success at an earlier age (reproductive senescence), resulting in different fitness costs. Environmental factors may affect this resource allocation and, indirectly, the pattern and intensity of senescence (Rodríguez‐Muñoz et al., 2019).

Adult body sizes in insects are partly genetically determined and partly influenced by environmental factors (e.g., larval and adult food availability or weather conditions); therefore, continuous monitoring and repeated measurements of phenotypic traits throughout an individual's life are required to study phenotypic senescence in natural populations. This is challenging in insects due to their small body size, high mobility, and cryptic life cycle (Nussey et al., 2008). The only study we found is Rodríguez‐Muñoz et al. (2019), in which they observed that the singing activity of male field crickets declined with age under natural conditions in five out of nine study years. In case of butterflies, decreasing thorax and abdomen mass with age has been demonstrated under laboratory conditions (Karlsson, 1994; Norberg & Leimar, 2002; Stjernholm et al., 2005; Stjernholm & Karlsson, 2000). Adult body size of butterflies and its change over time are partly determined by the amount and quality of nutrients ingested during the larval and adult stages, by resource allocation within the body, and by nuptial gifts given by males to females during mating (Stjernholm & Karlsson, 2000). Nitrogen seems to be one of the limiting components for reproduction (e.g., egg production) in adult butterflies (Boggs, 1981; Mattson, 1980). Pollen‐consuming butterflies, like *Heliconius hecale*, are able to receive a sufficient amount of nitrogen through feeding, but nectar consumers, like *Pararge aegeria* or *Speyeria mormonia*, are not (Karlsson, 1994). In order to increase fecundity or survival (Karlsson, 1994, 1998), nectar consumers are able to allocate nitrogen (Karlsson, 1998; Stjernholm et al., 2005; Stjernholm & Karlsson, 2000) and other resources (Boggs, 1981) from the abdomen, resulting in an age‐related decline in abdomen mass, or from thoracic muscles (e.g., flight muscles) (Stjernholm et al., 2005). In some species, females were proven to be able to transfer resources from thorax into eggs (e.g., *Pieris napi*, Karlsson, 1998). Resource reallocation can influence the disintegration of thorax and abdomen, resulting in body mass loss and, ultimately, accelerated senescence.

Differences were found in the direction and amount of change in body and thorax mass between polyandrous and monandrous species (Bissoondath & Wiklund, 1995). Males of polyandrous species invest more nutrients into the spermatophore than monandrous Lepidoptera due to increased competition between males (9 Pieridae and 2 Saturniidae species: Bissoondath & Wiklund, 1995; *Heliconius* spp.: Karlsson, 1995); therefore, females of monandrous species may utilize more nutrients from muscle breakdown (Stjernholm et al., 2005). However, in two (*Polygonia c‐album* and *Aglais urticae*) out of four strongly polyandrous species, Stjernholm et al. (2005) failed to reveal the relationship between the reserves used from thorax and the degree of polyandry among males, probably due to the deterioration of flight muscles that may affect flight performance (Ahman & Karlsson, 2009; Stjernholm et al., 2005).

In case of females, maintaining flight ability could be important to search for suitable sites for egg laying. Despite these, among insects, butterflies may benefit the most from using flight muscles as resources for reproduction (Stjernholm et al., 2005). Trade‐off between energy invested in survival or in reproduction seems to influence the evolution of life histories (Lemaître et al., 2015) and can be different between sexes. Different resource allocation patterns between the sexes may result in a steeper decrease in females' age‐related body and thorax mass loss compared with males (Karlsson, 1994; Norberg & Leimar, 2002; Stjernholm & Karlsson, 2000).

Most of the above‐mentioned studies were conducted in laboratory conditions and/or in a controlled environment and lasted only 1–3 years. Fitness components (Fry, 2008) and resources that determine body sizes and senescence are all affected by the experienced environmental conditions, which may vary seasonally (e.g., Alcock, 1984; Evans, 2000). Seasonal fluctuations of body size are mostly affected by developmental temperature (e.g., David et al., 1997), resource availability (e.g., Gibbs et al., 2012; Lehmann et al., 2006), or stressful conditions such as desiccation (e.g., Colinet et al., 2007; Lighton et al., 1994), intraspecific competition (e.g., Heinrich & Bartholomew, 1979), or predation intensity (e.g., Nylin & Gotthard, 1998). All these imply that studies conducted in natural populations may better reflect natural processes. Furthermore, laboratory populations are often genetically different from their conspecifics in the field due to genetic drift or adaptation to laboratory conditions (Matos & Avelar, 2001), potentially resulting in differences in aging (Kenyon, 2005; Kirkwood & Austad, 2000). Even genetically similar wild and captive groups of a population may show different degrees of senescence (Kawasaki et al., 2008). In addition, studies under controlled circumstances did not explain the variance in intra‐ and interspecific longevity and aging, nor how environmental variance affects the rate of aging (Flatt et al., 2013).

Senescence shows high variability among years in natural circumstances (Rodríguez‐Muñoz et al., 2019) as individuals have to face different environments each year. As senescence is a within‐individual process, we shall conduct long‐term studies (Rodríguez‐Muñoz et al., 2019) to control for between‐individual heterogeneity, which can mask within‐individual senescence (van de Pol & Verhulst, 2006). Longitudinal studies of identified individuals provide one of the best ways of detecting senescence in the wild (Nussey et al., 2008), but long‐term studies on body size variation in insect populations are scarce, both in the lab and in the wild.

We investigated the decline of body size with age in a natural population of the Clouded Apollo butterfly (*Parnassius mnemosyne* (Linnaeus, 1758)) over seven consecutive years. We measured body mass and thorax width, which can change during individuals' lifespan, and wing length, which does not change with age. Wing length is a widely used proxy of flight capacity in butterflies (Sekar, 2012), thus likely related to individual fitness.

We aimed to test whether (i) body mass and thorax width declined with age (phenotypic senescence), (ii) this decline was linear or polynomial, (iii) body size and its change with age differed between sexes, (iv) the date of first capture of an individual was related to its body size and the rate of senescence, and (v) wing length was correlated with body size and phenotypic senescence. We also attempted to distinguish the within‐subject and between‐subject effects of age and the date of first capture because individuals were measured at different ages and the date of first capture showed quite different distributions among years (van de Pol & Wright, 2009).

## 2 MATERIALS AND METHODS

### 2.1 Study species and site

Clouded Apollo butterfly is widespread in the Western Palearctic realm (van Swaay et al., 2012). In Central Europe, it inhabits woodland clearings and meadows, rich in flowering plants and open sunny areas, surrounded by woods (Weiss, 1991). This protandrous, univoltine species' flight period runs from late April to the beginning of June in Hungary. Adult Clouded Apollos spend plenty of time on feeding (Szigeti et al., 2018). Males search females by patrolling, and during mating, they may produce a large, presumably costly sphragis on the females' copulatory orifice (Vlašánek & Konvička, 2009). Mated females lay eggs singly on leaf litter or grass near *Corydalis* sp. patches. It overwinters as egg (Bergström, 2005), caterpillars hatch in early spring, and feed exclusively on *Corydalis* species.

Field work was conducted between 2014 and 2020 at Hegyesd, a 0.5‐hectare meadow (47°45′22.7″ N, 19°2′53.4″ E, at 295 m a.s.l.) in the Visegrádi‐hegység, Central Hungary. Insect‐pollinated flowering plants were heterogeneously distributed all over the meadow, surrounded by oak wood (*Quercus cerris*).

### 2.2 Sampling method

Mark‐release‐recapture (MRR) was used to sample the population. Field work covered the whole flight period every year. Sampling started when the first Clouded Apollo adults appeared and lasted until the last individual was on the wing. Data collection was conducted by 2–5 people every day during the flight period, as weather allowed. For the survey, observers followed the same routes, which had been systematically distributed in the meadow to reduce trampling (Szigeti et al., 2016). Butterflies were captured with butterfly nets and were marked with a unique color code and a number; the code was placed ventrally on the transparent tips of both forewings with Edding® paint markers; the number was written on the ventral side of both hindwings with Edding® permanent markers. Color codes could be seen from both sides of the wing.

We surveyed the meadow several times a day and tried to catch all unmarked Clouded Apollos. As it is a small, closed population (Table 1), we assumed that butterflies were captured soon after their eclosion, and their detectability did not vary among individuals and through time. Each year >90% of the individuals were captured and measured at least once (Zorkóczy, 2020).

**TABLE 1:** Dates, duration (days), and the number of marked individuals in each flight period.

Year	First day	Last day	Duration	*N*_males_	*N*_females_	*N*_individuals_
2014	2014‐04‐17	2014‐05‐26	40	123	84	207
2015	2015‐04‐26	2015‐05‐30	35	92	84	176
2016	2016‐04‐22	2016‐06‐03	43	106	88	194
2017	2017‐04‐25	2017‐05‐29	35	102	87	189
2018	2018‐04‐29	2018‐05‐24	26	155	116	271
2019	2019‐04‐21	2019‐06‐04	45	120	83	203
2020	2020‐04‐21	2020‐05‐21	31	41	34	75

Between 2014 and 2016, body mass measurements were performed every third day. In further years, it was measured at first capture, along with thorax width and wing length. We attempted to recapture all marked individuals to repeat body mass and thorax width measurements every third day. We measured body mass with a Mettler‐Toledo, NewClassic MF JS303G scale (Mettler‐Toledo AG, Laboratory & Weighing Technologies) with 1 mg precision. Thorax width was measured twice on each occasion with calipers to 0.1 mm. Wing length was the average length of both forewings measured from base to the apex with a commercial plastic ruler (2014–15; resolution: 1 mm) or a printed ruler (2016–20; resolution: 0.2 mm). If we measured a variable twice in a single measurement session, we used averages for all analyses. After marking and measuring, butterflies were instantly released. Clouded Apollo is a relatively robust species, which allows repeated measurements of body sizes without apparent harm.

Handling and the associated measurements were done by JK, except for body mass that was measured by several people.

Field work was licensed by the Hungarian nature conservation authorities: KTVF: 31430/2014.

### 2.3 Statistical analysis

We defined “age” as the number of days elapsed from the first capture of an individual, which was a minimum estimate for the real age. “Mean (age)” was the average of all age data at the time of measurements of an individual. “First capture” variable was the day of the flight period when an individual was captured for the first time, whereas “mean (first capture)” was the annual average of the days of first captures.

Data from the seven study years were pooled and analyzed within one model. First, we built a linear mixed‐effects model for each response variable (body mass and thorax width) including age, age^2^, mean (age), mean (age^2^), first capture, mean (first capture), and wing length, the interactions between these variables and sex, and the interactions between wing length and age and between first capture and age. Individual butterfly identifiers nested in year were used as hierarchical random factors (Table 2). Body mass was log‐transformed to improve model fit and to control for initial body mass.

**TABLE 2:** Full and most supported models' specifications

	Response variable	Explanatory variables	Random term
Linear mixed‐effects model (full model)	Log (Body mass)	sex × (age + mean age + age^2^ + mean_age^2^ + first capture + mean first capture + wing) + (age:wing) + (age:first capture)	random = ~1|year/id *or* random = ~age|year/id
	Thorax width	sex × (age + mean age + age^2^ + mean_age^2^ + first capture + mean first capture + wing) + (age:wing) + (age:first capture)	random = ~1|year/id *or* random = ~age|year/id
Most supported models	Log (Body mass)	sex × age^2^ + wing + age × first capture	random = ~age|year/id
	Thorax width	sex × age + mean age + age^2^ + first capture + mean age + wing length	random = ~age|year/id

*Note*: Mean_age^2^ is the average of the square of age variable.

Age^2^ was used to model a nonlinear relationship between age and body size. “Mean (age)” and “mean (age^2^)” were the averages of all age and age^2^, respectively, data at the time of measurements of an individual. These “mean variables” enabled us to distinguish the within‐subject and between‐subject effects (van de Pol & Wright, 2009). Butterflies not only had different body sizes but also their age at measurement showed high variation. By using “mean (age)” and mean (age^2^), we could test whether body size declined with age (within‐individual effect) and whether individuals measured at an older age had smaller body size (between‐individual effect). Distribution of “first capture” was different among years, and “mean (first capture)” is correlated with the length of the flight period (see variation in flight period length in Table 1). By using “mean (first capture)” as a covariate, we could also test whether body size variation among years was related to the variation in flight period length. The relationship between body mass, thorax width, and wing length, and the effect of wing length on body mass and thorax width decline with age were also tested with these models. We built these “full” models with both random slope and random intercept structures. Based on AICc values, the random slope models were more supported for both response variables. Then, we removed all nonsignificant (*p* > .05) interactions from the full model and then applied an all‐combination automated AICc‐based model selection from this reduced model using the “dredge” function of the “MuMIn” package (Bartoń, 2020). The preselected/reduced model for body mass included 11 explanatory variables, and 520 models were tested. For thorax width, 13 explanatory variables were included and 1073 models were tested. Finally, parameter estimates of models with ΔAICc < 4 were averaged using the “model.avg” function of the “MuMIn” package. Model diagnostics of the most supported models (Table 2) were checked by inspecting residual plots; the “VarCorr” function (“nlme” package) was used to calculate the proportion of variance explained by the random term.

We also compared the initial mean values for body mass and thorax width between individuals with only one or repeated (at least two) measurements.

All statistical analyses were done using packages “nlme” (v3.1‐152; Pinheiro et al., 2007) and “MuMIn” (v1.43.17; Bartoń, 2020) in R 3.6.1 (R Core Team, 2021).

## 3 RESULTS

Between 2014 and 2020, we measured body mass at least one of 1191, and at least twice of 826 (69.35%) Clouded Apollos (Table 3). We measured the thorax width of 1312 individuals and of 746 (56.86%) individuals repeatedly. The largest proportion of individuals with repeated measures was in 2019 when 82.27% and 80.79% of captured butterflies had repeated body mass and thorax width measurements, respectively. Individuals with the most measurements of body mass (7×, 8×, or 9×) occurred in 2016 and 2019, till in case of thorax mass in 2017 and 2019.

**TABLE 3:** Number of measured individuals according to the number of measurements in each year

	No. of individuals measured				No. of individuals with repeated measurements (without overlapping)								
	All	Single measure	Repeated measures	% repeatedly measured	1	2	3	4	5	6	7	8	9
Body mass													
2014	167	62	105	62.87	62	55	32	12	4	2			
2015	138	62	76	55.07	62	32	23	14	7				
2016	178	32	146	82.02	32	36	36	27	24	12	10	1	
2017	160	53	107	66.88	53	52	27	20	8				
2018	271	101	171	63.10	100	81	59	19	10	2			
2019	203	36	167	82.27	36	42	48	34	22	9	9	1	2
2020	74	20	54	72.97	20	22	18	11	2	1			
Sum	1191	366	826	69.35									
Thorax width													
2014	205	141	64	31.22	141	54	8	2					
2015	176	101	75	42.61	101	60	8	6	1				
2016	193	121	72	37.31	121	40	21	6	3	2			
2017	189	38	151	79.89	38	46	49	24	18	11	2	1	
2018	271	106	165	60.89	106	75	58	19	9	4			
2019	203	39	164	80.79	39	45	41	33	24	10	9	2	
2020	75	20	55	73.33	20	19	19	11	5	1			
Sum	1312	566	746	56.86									

The largest mean initial body mass was in 2019 for females (0.232 g) and in 2020 for males (0.194 g). The smallest was in 2015 for females (0.213 g) and in 2018 for males (0.171 g). The largest mean initial thorax width for both sexes was detected in 2016 (2.131 mm and 2.115 mm for females and males, respectively) and the smallest in 2014 (1.900 mm and 1.851 mm for females and males, respectively) (Table 4).

**TABLE 4:** Annual mean (±SE) initial body mass and thorax width (maximum values among years are green, minimum values are orange).

Year	Mean initial body mass		Mean initial thorax width	
	Female (± SE)	Male (± SE)	Female (± SE)	Male (± SE)
2014	0.226 (± 0.005)	0.179 (±0.003)	1.900 (± 0.019)	1.851 (± 0.014)
2015	0.213 (± 0.006)	0.173 (± 0.004)	2.041 (± 0.024)	1.985 (± 0.017)
2016	0.227 (± 0.004)	0.186 (± 0.003)	2.131 (± 0.018)	2.115 (± 0.017)
2017	0.226 (± 0.005)	0.186 (± 0.004)	2.085 (± 0.019)	2.043 (± 0.015)
2018	0.214 (± 0.003)	0.171 (± 0.002)	1.905 (± 0.015)	1.885 (± 0.014)
2019	0.232 (± 0.005)	0.191 (± 0.004)	2.042 (± 0.016)	1.973 (± 0.013)
2020	0.219 (± 0.004)	0.194 (± 0.004)	2.026 (± 0.022)	2.028 (± 0.018)

Body mass and thorax width at first measurements were significantly larger in repeatedly measured individuals (mass: 0.205 ± 0.042 g; thorax: 2.023 ± 0.172 mm; mean ± SD) than in individuals measured only once (mass: 0.187 ± 0.038 g; thorax: 1.943 ± 0.193 mm; mean ± SD) (mass: *F*_7,1183_ = 48.787, *p* < .001; thorax: *F*_7,1304_ = 78.852, *p* < .001). In repeatedly measured individuals, initial body mass was significantly higher for females in all years.

The longest flight period was 45 days in 2019, while the shortest with 26 days was in 2018 when the most Clouded Apollos (271 individuals) occurred (Table 1).

**FIGURE 1:**
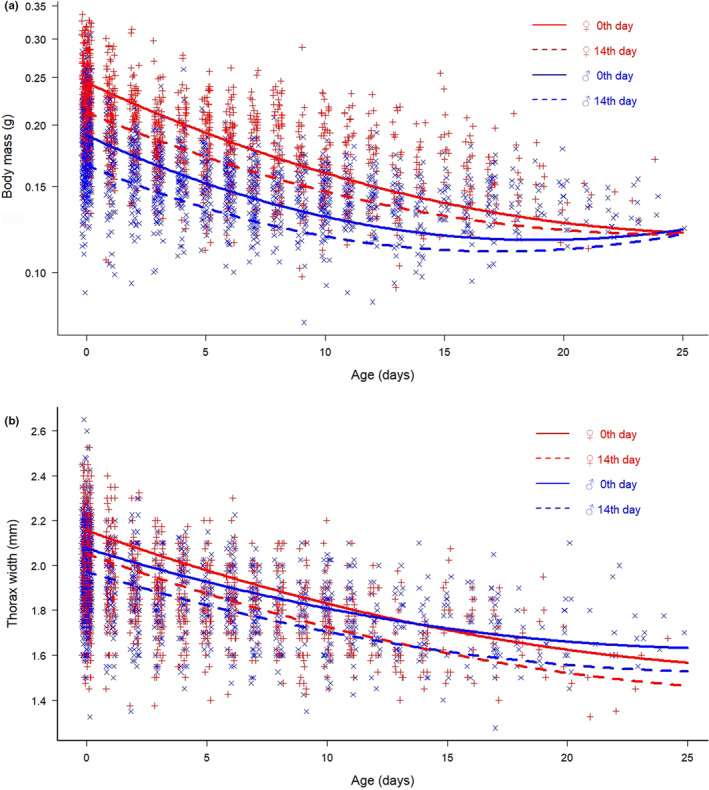
Body mass (a) and thorax width (b) change with age. Symbols represent the individuals' measurements (red + = females; blue × = males). Dots are slightly jittered along the *x*‐axis for better visibility. Lines represent the estimated relationship between age and body size for the two sexes and of an individual with average wing length (31.4 mm), with first capture = 0th day (solid lines) or first capture = 14th day (dashed lines) of the flight period.

We found a significant decline in body mass with increasing age in both sexes. This relationship was nonlinear, body mass decreased slower at an older age. Males had significantly lower initial body mass than females, but males' body mass declined slower at an older age (the sex × age^2^ interaction was significant); thus, the two sexes had approximately similar body mass by the end of their life. Butterflies with longer wing length had higher initial body mass, but wing length did not affect body mass decline. Body mass also significantly declined with the date of first capture, i.e., individuals captured and marked later in the flight period were smaller, but their body mass also declined slower (Figure 1, Tables 5 and 6).

**TABLE 5:** The results of model selection for log (body mass) for linear mixed‐effects model with random slope.

Model specification	AICc	ΔAICc	Df	logLik
log (body mass) ~ sex × age^2^ + age × first capture + wing, random = ~age|year/id	−4279.6	0.00	15	2154.858
log (body mass) ~ sex × age^2^ + mean age + age × first capture + wing, random = ~age|year/id	−4279.4	0.17	16	2155.781
log (body mass) ~ sex × age^2^ + age × first capture + wing + mean (first capture), random = ~age|year/id	−4278.8	0.71	16	2155.512
log (body mass) ~ sex × age^2^ + mean age + age × first capture + wing + mean (first capture), random = ~age|year/id	−4277.0	2.55	17	2155.604
log (body mass) ~ sex × age^2^ + age × first capture + mean age^2^ + wing, random = ~age|year/id	−4270.4	9.15	16	2151.291
log (body mass) ~ sex × age^2^ + age × first capture + mean age^2^ + wing + mean first capture, random = ~age|year/id	−4268.3	11.24	17	2151.262

**TABLE 6:** The results of model averaging for log (body mass) linear mixed‐effects model (subset = ΔAICc < 4).

Trait	Model‐averaged coefficients (full average)	Estimate	SE	Adjusted SE	*z*‐Value	*p*‐Value
Log (Body mass)	(Intercept)	−2.9810	.1272	.1273	23.4180	<.001
	Age	−0.0519	.0019	.0019	26.8430	<.001
	Age^2^	0.0009	.0001	.0001	13.0420	<.001
	First capture	−0.0103	.0008	.0008	13.0310	<.001
	Sex (male)	−0.2482	.0078	.0078	31.6940	<.001
	Wing	0.0497	.0027	.0027	18.3860	<.001
	Age:first capture	0.0005	.0001	.0001	6.5300	<.001
	Age^2^:sex (male)	0.0004	.0001	.0001	7.2820	<.001
	Mean (age)	0.0019	.0023	.0023	0.7890	.430
	Mean (first capture)	0.0057	.0081	.0083	0.6870	.492

After model averaging, the coefficients of mean (age), mean (age^2^), and mean (first capture) were not significant. In this kind of model parametrization, these coefficients are the differences between the within‐subject and between‐subject effects (van de Pol & Wright, 2009), thus we can conclude that these effects did not differ significantly. This means that body mass declined significantly with age and that individuals measured at (on average) an older age had lower body mass. We note that the coefficient of mean (first capture) had a relatively large positive value, and it was significant in those models where it was included (Tables 5 and 6), suggesting that the between‐subject effect was positive. This means that within each year, individuals captured later had lower body mass, but average body mass was higher in those years when the flight period length was longer (i.e., mean (first capture) was higher). This, however, does not seem to be a very strong relationship.

Thorax width also declined with age nonlinearly in both sexes, but the rate of decline was slightly higher in females. Thorax width was smaller in males than in females and it was positively related to wing length. Butterflies captured later during the season had smaller thorax width. Mean (age) proved to be significant, but its value was relatively low implying that within‐subject and between‐subject effects had similar directions (Figure 1, Tables 7 and 8). This means that thorax width significantly declined with age (within‐subject effect) and individuals measured at (on average) an older age had smaller thorax width (between‐subject effect).

**TABLE 7:** The results of model selection for thorax width for linear mixed‐effects model with random slope.

Model specification	AICc	ΔAICc	Df	logLik
Thorax width ~ sex × age^2^ + mean age + age × first capture + wing + age:sex, random = ~age|year/id	−3391.3	0.00	15	1710.758
Thorax width ~ sex × age^2^ + mean age + age × first capture + wing + mean (first capture) + age:sex, random = ~age|year/id	−3389.2	2.14	16	1710.697
Thorax width ~ sex × age^2^ + mean age + age × first capture + wing + age:first capture + age:sex, random = ~age|year/id	−3382.9	8.42	16	1707.559
Thorax width ~ sex × age^2^ + mean age + age × first capture + wing + mean (first capture) + age:first capture + age:sex, random = ~age|year/id	−3381.0	10.34	17	1707.613
Thorax width ~ sex × age^2^ + mean age + age × first capture + wing + mean (first capture) + age:sex + mean (first capture):sex, random = ~age|year/id	−3380.6	10.70	17	1707.429

**TABLE 8:** The results of model averaging for thorax width for linear mixed‐effects model (subset = ΔAICc < 4).

Trait	Model‐averaged coefficients (full average)	Estimate	SE	Adjusted SE	*z*‐Value	*p*‐Value
Thorax width	(Intercept)	0.7892	.1621	.1621	4.8680	<.001
	Age	−0.0386	.0020	.0020	19.7230	<.001
	Age^2^	0.0006	.0001	.0001	7.4410	<.001
	First capture	−0.0074	.0005	.0005	14.0630	<.001
	Mean (age)	0.0080	.0013	.0013	6.1730	<.001
	Sex (male)	−0.0751	.0079	.0079	9.5010	<.001
	Wing	0.0397	.0026	.0026	15.3840	<.001
	Age:sex (male)	0.0059	.0010	.0010	6.1410	<.001
	Mean (first capture)	0.0059	.0106	.0109	0.5450	.585

The random factors explained a high proportion of total variance in the most supported models for body mass (~70%) and thorax width (~62%) (Table 9).

**TABLE 9:** Proportion of variance of random terms in linear mixed‐effect models

Trait	Random effects	Fixed effects	Variance	Proportion of variance (%)
Body mass	Id	Intercept	0.0118	50.40
		Age	0.000035	0.15
	Year	Intercept	0.0045	19.22
		Age	0.00001	0.04
	Residual		0.00707	30.19
Thorax width	Id	Intercept	0.0088	31.17
		Age	0.000019	0.07
	Year	Intercept	0.0086	30.46
		Age	0.000014	0.05
	Residual		0.0108	38.25

The highest proportion of variance was explained by the between‐individual differences in both variables. In case of body mass, this variation was much higher than between‐year variance, while for thorax width the between‐year variation was nearly the same as the between‐individual variation. These indicate that initial body mass had a larger between‐individual than between‐year variation, while initial thorax width had a similar variation between individuals and between years. The age effect showed much lower variation indicating that initial body mass and thorax width had much higher variation than the rate of aging.

## 4 DISCUSSION

We found a significant decrease in body mass and thorax width with age in both sexes, with high individual variation (Figure 1).

Variation and change in butterflies' body mass can be influenced by many factors. Body mass loss was related to water loss during desiccation in Heliconiinae butterflies; total body water differed among species and it was lower for females than males likely due to higher fat content (Mazer & Appel, 2001). In freshly emerged Woodland Brown butterflies (*Pararge aegeria*), (i) lipid reserves were higher than in the 2–3 days old indicating a decline in lipid reserves with aging in a laboratory study, and (ii) males with lower lipid reserves formed smaller spermatophores showing a less steep decline in body mass (Vande Velde et al., 2013). Oviposition or spermatophore transmission may also result in a change in body mass (Stjernholm & Karlsson, 2000). Clouded Apollo females may receive sphragis (mating plug) during mating (Vlašánek & Konvička, 2009), making up to 3%–5% of the measured female body mass (authors' unpublished data). This presumably causes body mass loss in males and it may also be a burden for females as flying with sphragis could be costly. In addition, post‐emergence body mass may be higher due to yet unreleased meconium.

Reserves used from abdomen and thoracic muscles may also cause decline, especially in thorax width. Resource allocation during adult and (possibly) larval stages can be affected by trade‐offs between survival and reproduction (Boggs, 2009). For instance, the nitrogen content of the flight muscles may be used for reproduction in Green‐veined White (*Pieris napi*) butterfly females (Karlsson, 1998), causing thorax mass decrease. However, using thoracic muscles as a resource may be restricted by flight performance (Stjernholm et al., 2005). This could be the case of Clouded Apollo males as they should patrol to find new mates.

We detected high individual variations in initial body size and minor variation in its change with age. Random slope models were more supported than random intercept models, indicating that the rate of body size decline with age has a non‐negligible individual variance. However, the random term of initial body sizes (intercept) explained a much larger proportion of the total variance (Table 9). Clouded Apollos spend a lot of time feeding (Szigeti et al., 2018). In our study population, individuals' body mass may vary ~10% daily with the timing of feeding versus other activities (flying; patrolling; mating; egg laying) that incur net weight loss (authors' unpublished data). These activities may cause detectable changes in body mass within a day and might explain the high individual variation.

We also observed sexual dimorphism in body size and the rate of change in body size with a faster decline in females. Both initial body mass and thorax width were larger in females. Body mass declined slower with age in males only at an older age (Figure 1). However, thorax width declined slower in males already at a younger age, thus above the age of ca. 14 days males had on average wider thoraces than females (Figure 1). The presence of such differences may be influenced by many circumstances and can be derived from the dissimilar physiology and behavior of the sexes. The accelerated decline may be caused by egg laying in females or by producing spermatophores and/or sphragides in males. Resource allocation can be different between the sexes, e.g., females may use more nutrients from muscle breakdown for reproduction than males, therefore experiencing a steeper decline (Stjernholm et al., 2005), but males may also invest into reproduction through the spermatophore, and in the case of the Clouded Apollo, through the sphragis (Vlašánek & Konvička, 2009). Flying is energetically costly (Dudley, 2002) and male butterflies often fly more than females (Popović et al., 2022). According to our field observations, Clouded Apollo males spend a lot of time patrolling to find mating partners, hence they possibly lose more water and reserves from their bodies than females. Moreover, as male reproductive success likely depends on flight ability more than females', there might be a strong selection against decomposing their thoracic muscles. In contrast, 69%–77% of females are sphragis‐bearing (authors' unpublished data), and flight costs might be higher for them than for those lacking sphragis or for males. We have no data on the frequency of feeding and the amount of nectar consumed, but it is possible that either males or females can compensate for the faster body mass loss with a higher feeding rate.

Body size of individuals newly appearing in the population showed a declining trend with the progress of the flight period. Clouded Apollos are protandrous, i.e., males emerge earlier in the flight period than females to maximize their reproductive success (Fagerström & Wiklund, 1982; Wiklund & Fagerström, 1977). A recent meta‐analysis in insects revealed that the direction and degree of sexual bimaturism and sexual size dimorphism are positively associated (Teder et al., 2021). This is partly supported by our results as females were larger than males in all years. Unlike in a laboratory study, here we could not control for environmental conditions (e.g., temperature, humidity, food availability, etc.) during larval development, therefore we can only speculate about the reasons underlying our results. Based on that larger body size usually means higher fecundity in insects (Honěk, 1993) and that later emergence has no apparent benefit, we suspect that there is a “growth race” among the larvae and individuals pupate upon reaching an optimum size. This optimum might be influenced by genetic factors as well as environmental constraints on development. Winners of the growth race, i.e., the early pupating and emerging individuals, must be soon followed by slower developers, otherwise they would not find mating partners. Thus, the slower developing individuals might be forced to pupate before reaching the body size of earlier individuals. Note that the classical life‐history theory typically predicts a decreasing reaction norm between optimal body size and age at maturity when the growth rate varies in different environmental conditions (Stearns & Koella, 1986).

Alternatively, Clouded Apollos appeared later in the flight period and may have hatched later from the eggs, so they may have had less time to develop. Later hatching can also be disadvantageous because aged host plants may have lower nutrient content (e.g., Mattson, 1980) and/or higher concentration of defense chemicals or physical defenses (Barton & Koricheva, 2015; Yang et al., 2020). Restricted amount or bad quality food may also cause prolonged larval development time (Gibbs et al., 2012) or smaller adult body size (Boggs & Freeman, 2005; Boggs & Niitepõld, 2016; Niitepõld & Boggs, 2022).

We revealed that butterflies with repeated measurements had significantly higher initial body mass and thorax width than those measured only once, i.e., we encountered, captured, and measured larger Clouded Apollos more often than the smaller ones. This is likely due to that butterflies appearing earlier in the flight period were larger and had a higher chance to be recaptured and re‐measured. Larger butterflies might also be more active (patrolling, flying, feeding, etc.), leading to higher detectability and/or better survival abilities, but further investigations are needed to clarify this phenomenon.

## 5 CONCLUSIONS

To our knowledge, this is the first study that revealed phenotypic senescence in a natural butterfly population, using in vivo measurements. We found large variations in initial body size and smaller in the rate of senescence among individuals. Females had larger initial body sizes but they declined faster than males, indicating that differential selection influences phenotypic senescence. Our results suggest that the rates of senescence and larval growth may be influenced by individually and annually varying environmental variables and genetic factors. Body size largely determines and senescence may also affect reproductive success in insects. Therefore, we recommend that laboratory studies in the future should aim to uncover the effects of weather conditions and resource availability on the rates of larval growth and adult senescence. Ultimately, a deeper understanding of these relationships would help us better predict the effects of current global environmental changes on the viability of insect populations.

## AUTHOR CONTRIBUTIONS

**Kata Pásztor:** Conceptualization (equal); data curation (equal); formal analysis (equal); investigation (equal); methodology (equal); resources (supporting); visualization (lead); writing – original draft (lead). **Ádám Kőrösi:** Conceptualization (equal); formal analysis (equal); investigation (equal); methodology (equal); project administration (lead); supervision (lead); writing – review and editing (equal). **Ádám Gór:** Data curation (equal); formal analysis (equal); investigation (equal); methodology (equal); resources (supporting); visualization (lead); writing – review and editing (supporting). **Viktor Szigeti:** Investigation (equal); methodology (equal); resources (supporting); writing – review and editing (supporting). **Flóra Vajna:** Investigation (equal); methodology (equal); resources (supporting); writing – review and editing (supporting). **János Kis:** Conceptualization (equal); investigation (equal); methodology (equal); project administration (lead); resources (lead); supervision (lead); writing – review and editing (equal).

## CONFLICT OF INTEREST

All authors declare that they have no conflict of interest.

## Data Availability

Data available from the Dryad Digital Repository: 10.5061/dryad.jq2bvq8bp.
